# C-Reactive Protein Suppresses the Th17 Response Indirectly by Attenuating the Antigen Presentation Ability of Monocyte Derived Dendritic Cells in Experimental Autoimmune Encephalomyelitis

**DOI:** 10.3389/fimmu.2021.589200

**Published:** 2021-03-25

**Authors:** Zhi-Yuan Shen, Yi Zheng, Maggie K. Pecsok, Ke Wang, Wei Li, Min-Jie Gong, Feng Wu, Lin Zhang

**Affiliations:** ^1^ Department of Biochemistry and Molecular Biology, School of Basic Medicine, Xi’an Jiaotong University, Xi’an, China; ^2^ School of Medicine, University of Electronic Science and Technology of China, Chengdu, China; ^3^ Departments of Neurology and Immunology, School of Medicine, Yale University, New Haven, CT, United States; ^4^ MOE Key Laboratory of Cell Activities and Stress Adaptations, School of Life Sciences, Lanzhou University, Lanzhou, China; ^5^ Department of Otolaryngology Head and Neck Surgery, The Second Affiliated Hospital of Xi’an Jiaotong University, Xi’an, China; ^6^ Center of Teaching and Experiment for Medical Post Graduates, School of Basic Medicine, Xi’an Jiaotong University, Xi’an, China

**Keywords:** experimental autoimmune encephalomyelitis, C-reactive protein, monocyte derived DC, Fc*γ*R2B, Th17 response

## Abstract

Experimental autoimmune encephalomyelitis (EAE) is a classical murine model for Multiple Sclerosis (MS), a human autoimmune disease characterized by Th1 and Th17 responses. Numerous studies have reported that C-reactive protein (CRP) mitigates EAE severity, but studies on the relevant pathologic mechanisms are insufficient. Our previous study found that CRP suppresses Th1 response directly by receptor binding on naïve T cells; however, we did not observe the effect on Th17 response at that time; thus it remains unclear whether CRP could regulate Th17 response. In this study, we verified the downregulation of Th17 response by a single-dose CRP injection in MOG-immunized EAE mice *in vivo* while the direct and indirect effects of CRP on Th17 response were differentiated by comparing its actions on isolated CD4^+^ T cells and splenocytes *in vitro*, respectively. Moreover, the immune cell composition was examined in the blood and CNS (Central Nervous System), and a blood (monocytes) to CNS (dendritic cells) infiltration pathway is established in the course of EAE development. The infiltrated monocyte derived DCs (moDCs) were proved to be the only candidate antigen presenting cells to execute CRP’s function. Conversely, the decrease of Th17 responses caused by CRP disappeared in the above *in vivo* and *in vitro* studies with Fc*γ*R2B^−/−^ mice, indicating that Fc*γ*R2B expressed on moDCs mediates CRP function. Furthermore, peripheral blood monocytes were isolated and induced to establish moDCs, which were used to demonstrate that the antigen presenting ability of moDCs was attenuated by CRP through Fc*γ*R2B, and then NF-*κ*B and ERK signaling pathways were manifested to be involved in this regulation. Ultimately, we perfected and enriched the mechanism studies of CRP in EAE remission, so we are more convinced that CRP plays a key role in protecting against EAE development, which may be a potential therapeutic target for the treatment of MS in human.

## Introduction

Multiple Sclerosis (MS) is an autoimmune disease in the human brain and spinal cord, referred to as central nervous system (CNS), that is characterized by immune cell infiltration, neuroinflammation, demyelination, and axonal damage (1, 2). Although the exact etiology of MS is unclear, it is generally considered to involve environmental, genetic, and immunological factors, and the immunopathology especially has been established and accepted for the last four decades (3–6). Experimental autoimmune encephalomyelitis (EAE) is a widely used murine model of MS, and a plethora of studies have shown that myelin specific CD4^+^ T cells have a crucial role in the induction of EAE (7, 8).

CRP is an evolutionarily conserved pentamer plasma protein, and its plasma concentration increases dramatically as high as 1,000-fold during tissue injury and infection (9, 10). In the clinical setting, CRP is generally recognized as a non-specific inflammatory marker. As an innate immune molecule, CRP usually recognizes the Fc receptors to activate the classical pathway of complement and opsonize the phagocytosis of phagocytes in host defense (11, 12). Nevertheless, accumulating evidence indicates that CRP also plays an important role in some autoimmune diseases, such as systemic lupus erythematosus (SLE), rheumatoid arthritis (RA) and EAE (13–15). Among these, EAE has been studied most extensively in recent years, and it is reported that CRP has a protective role in MOG/MBP immunized EAE with human CRP transgenic (*hCRP*tg) mice or single dose human CRP injection (13, 16). However, the anti-inflammatory mechanism of CRP in EAE is still unclear and needs to be further investigated. IFN-*γ*-producing helper T cells (Th1 cells) and IL-17-producing helper T cells (Th17 cells) are crucial mediators in both EAE and MS (17, 18). We previously found that CRP suppresses Th1 response by direct receptor binding to naïve T cells, and that Th1 response also was reduced in MOG-immunized EAE model after CRP treatment (16), whereas we did not detect changes in IL-17 or ROR*γ*t when CD4^+^ T/naïve T cells were incubated directly with CRP at that time, so we wanted to investigate whether CRP regulates Th17 response in EAE pathogenesis.

The specific interactions between CRP and T cells are poorly studied, with initial reports saying that CRP could bind with T cells and thereby mediate the effective function in mid-1970s, but later this binding was denied by the same group (19, 20). Although we proved the direct binding of naïve T cells and pentamer CRP, this binding was not associated with Th17 regulation. Therefore, we moved our attention to antigen presenting cells (APCs), which usually express CRP’s three traditional receptors (21, 22). APCs internalize extracellular antigens and present MHC-I/II binding antigen fragments to T cells, generating antigen-specific CD4^+^ T cells (23). Some existing studies reported that CRP participates in CD4^+^ T cell responses through APCs, including monocytes, dendritic cells (DCs), and macrophages (24–26). However, we still don’t know which type of APC mediates the CRP’s function in EAE. Even though the Szalai group reported recently that CRP impairs DC maturation and function, thereby affecting CD4^+^ T cell responses in EAE development (27), it is still unclear why they focused on DCs directly and where did these DCs come from during EAE onset. Moreover, it is only a theoretical possibility that CRP affects T cell responses by inhibiting APC maturation, as only T cell proliferation was assessed in previous studies, and the CD4^+^ effector T cells have never been detected so far (24, 27). In this paper, we focus on the effects of CRP on Th17 response, and Th1, Th2, and Treg are assessed for the first time. Furthermore, we will investigate the moDCs’ antigen presentation ability regulated by CRP, something which remains controversial (24, 27, 28), as well as the signaling pathways involved in altering the antigen presenting molecules by CRP.

With this paper, we demonstrated that CRP regulates Th17 response indirectly by influencing the antigen presenting ability of moDCs though Fc*γ*R2B. In addition, the mechanisms by which CRP inhibits EAE development are further completed and elaborated. These findings not only provide profound insight into the contribution of CRP in host defense, but also put forward new ideas and potential targets for the intervention of autoimmune diseases.

## MATERIALS and METHODS

### Reagents

Human pentamer CRP, purified from ascites (purity>99%), was purchased from the Binding Site (BP300.X, Birmingham, United Kingdom). Generally, CRP was treated to further purification with immobilized p-Aminophenyl Phosphoryl Choline (Cat: 20307, Lot: RH237939,Thermo Fisher Scientific, Waltham, MA, USA) and a Detoxi-Gel column (20344, Thermo Fisher Scientific) packed with Polymixin B ligand immobilized on beaded affinity resin to bind and extract endotoxins from protein samples, as has been described in our previous studied (29, 30). Antibodies to p-STAT1 (Cat: 9167s, Lot: 4), STAT1 (Cat: 9172s, Lot: 25), STAT3 (Cat: 4904s, Lot: 7), p-STAT3 (Cat: 9145s, Lot: 34), p-ERK1/2 (4370s, Cat: 2), ERK1/2 (Cat: 4695s, Lot: 1) and NF-*κ*B p65 (Cat: 8242s, Lot: 1) were purchased from Cell Signaling Technology (Danvers, MA, USA). Anti-mouse CD25 APC (Cat: 17-02510-82, Lot: 4276862), anti-mouse MHC-II APC(I-A/I-E) (Cat: 17-5321-81, Lot: 1991457), anti-mouse CD86 APC (Cat: 17-0862-81, Lot: 1984132), anti-CD3 mAb (Cat: 16-0031-85, Lot: 4349473), anti-CD28 mAb (Cat: 16-0281-85, Lot: 1974623), antibodies to ROR*γ*t (Cat: 14-6981-82, Lot: 1936480), T-bet (Cat: 14-5825-82, Lot: 2012147) and Mouse IL-17AF ELISA Set (Cat: 88-8711-88, Lot: 4291151) were obtained from ebioscience (San Diego, CA, USA). Anti-mouse CD4 FITC (Cat: 553046, Lot: 5027567), anti-mouse IFN-*γ* PerCP-Cy5.5 (Cat: 560660, Lot: 5244738), anti-mouse IL-17A PE (Cat: 559502, Lot: 8071502), anti-mouse CD11b PE (Cat: 557397, Lot: 9023691), anti-Mouse CD45 APC (Cat: 559864, Lot: 8277680), anti-Mouse CD11c FITC (Cat: 557400, Lot: 8060996), anti-Mouse CD45R/B220 FITC (Cat: 553087, Lot: 8152878), Mouse IFN-*γ* ELISA Set (Cat: 555138, Lot: 7192700), Mouse IL-10 ELISA Set (Cat: 555252, Lot: 6154834), BD Pharm lyse™ (Cat: 555899, Lot: 8250695), Fixation/Permeabilization Solution Kit with BD GolgiPlug™ (Cat: 555028, Lot: 5261614) were purchased from BD Biosciences (San Jose, CA, USA). Anti-mouse PD-L1 APC (Cat: 124311, Lot: B277024) and anti-mouse OX40L APC (Cat: 108811, Lot: B274358) were purchased from Biolegend (San Diego, CA, USA).

### Animals

Wild-type mice (strain C57BL/6) were from the Experimental Animal Center of Xi’an Jiaotong University. CRP^−/−^mice were generated through Shanghai Model Organisms Co. Ltd (Shanghai, China). Fc*γ*R2B^−/−^ mice were purchased from Jackson Lab (Bar Harbor, Maine, USA). All mice were housed in the same vivarium at constant humidity (60 ± 5%) and temperature (24 ± 1°C) with a 12-h light/dark cycle. All procedures for the use of animals were approved by the Animal Ethics Committee of Xi’an Jiaotong University.

### Induction and Evaluation of EAE

Experimental autoimmune encephalomyelitis (EAE) was induced as we described previously (16). Briefly, 10–12 week old female mice were immunized subcutaneously with 200 µg myelin oligodendrocyte glycoprotein (MOG, MEVGWYRSPFSRVVHLYRNGK) peptide 35–55 (≥99% purity, Shanghai Science Peptide Biological Technology, Shanghai, China) in complete Freund’s adjuvant containing 4 mg *Mycobacterium tuberculosis* strain H37Ra (Cat: 7027, Lot: 180226, Chondrex, Redmond, WA, USA). On days 0 and 2, immunized mice received an intraperitoneal injection of 200 ng pertussis toxin (PTX, Cat: 181, Lot: 181238A1, List Biological Labs, CA, USA). On day 2, immunized mice received a single intraperitoneal injection of 200 μg human CRP or control buffer, and then the development of EAE was monitored daily. Neurological impairment was quantified daily on an arbitrary clinical scale: 0, asymptomatic; 1, decrease of tail tonicity; 2, limp tail and weakness of hind limb; 3, limp tail and partial hind limb paralysis; 4, limp tail, complete hind limb and partial foreleg paralysis; 5, moribund (31, 32). The splenocytes were isolated at the peak of EAE symptoms and re-stimulated *in vitro* with 50 μg/ml MOG peptide 35–55. Flow cytometry and ELISA determined intracellular cytokines and secreted cytokines respectively.

### Splenocytes and CD4^+^ T Cells’ Separation

Splenocytes were directly obtained from the spleens after removing the red cells by BD Pharm lyse™. CD4^+^ T cells were purified from the spleens using MACS kits (Cat: 130-049-201, Miltenyi Biotec, Bergisch Gladbach, Germany). The splenocytes and CD4^+^ T cells were cultured in RPMI 1640 medium (Cat: 11875-093, Gibco) containing 10% fetal bovine serum (BISH5400, BI), 1% penicillin/streptomycin, 50 uM 2-mercaptoethanol and were maintained in a humidified incubator with 5% CO2 at 37°C overnight. The cells were treated in 96-well culture plates (2.5 × 10^5^ cells in 300 ul per well) with plate-bound anti-CD3 (2 μg/ml, immobilized overnight at 4°C) and fluid phase anti-CD28 (2 μg/ml), in the presence or absence of CRP (100 μg/ml), and then collected after 24 h for mRNA detection and 72 h for protein detection.

### Th Cell Differentiation

The splenocytes and CD4^+^ T cells were obtained and cultured 3 days under Th1-polarizing conditions (10 ng/ml mIL-2, 20 ng/ml mIL-12p70, and 10 mg/ml anti-IL-4 mAb) or Th17-polarizing conditions (25 ng/ml IL-6, 5 ng/ml TGF-*β*, 20 ng/ml IL-1*β*, 10 μg/ml anti-IL-4 mAb and 10 μg/ml anti-IFN-*γ* mAb) (33, 34). The cells were transferred to a 24-well plate for an additional 2 days’ expansion. At the end of the culture, PMA (20 ng/ml), ionomycin (1 mg/ml), and BD GolgiPlug protein transport inhibitor containing brefeldin A were added for 4 h incubation. Cells were then collected for Flow Cytometry analysis.

### Immune Cells’ Isolation From Peripheral Blood and CNS

Mice were anesthetized by tribromoethanol, and the blood was collected from the eyeballs of mice. Mice were then perfused through the left ventricle with cold PBS. Brains and spinal cords were dissected and grounded through a cell strainer (70 μm), then re-suspended in 3 ml 30% percoll (GE, Cat: 17-0891-01, Lot: 1024671), and layered slowly on top of the 10 ml 70% percoll. After 30 min centrifugation at 800×g at 18°C (Acceleration,3; Deceleration,2), the layer of debris from the top of the tube was gently removed. Mononuclear cells were collected from the 70 to 30% interphase into a clean conical tube with 8 ml of 1× HBSS and washed for three times by centrifugation for 10 min at 500×g at 18°C. Finally, the cells were re-suspended in culture medium for flow cytometry.

### moDCs’ Generation, Culture, and Activation

Mouse moDCs were isolated from peripheral blood mononuclear cells (PBMCs) of 6–8 weeks old mice. Briefly, PBMCs were obtained through Ficoll gradient centrifugation, and after a 2 h adherence in 75 cm^2^ flasks, the non-adherent cells were removed and the adherent cells were cultured in RPMI 1640 containing 10% fetal bovine serum, 20 ng/ml GM-CSF (Cat: 415, Lot: BJ2519024, R&D), 10 ng/ml IL-4 (Cat: 550067, Lot: 8151542, BD Biosciences). The culture medium was replaced 3/4 on days 2 and 4. On day 6, the cells were harvested and seeded (1 × 10^5^/ml) in 24-well plates with or without CRP (100 μg/ml), and LPS (1 ug/ml) was added for moDC maturation on day 8. The moDCs were then harvested for flow cytometry and western blot on day 9.

### Real-Time PCR and Western Blot

Total RNA was extracted with RNAiso Plus reagent (9190, Takara, Shiga, Japan), and reverse transcribed using a Prime Script RT Master Mix Kit (RR036A, Takara). The target genes were quantified by quantitative real-time PCR using RealStar Green Power Mixture (A311, Genestar, Beijing, China) in a StepOne Plus real-time PCR system (Thermo Fisher Scientific).The primer sequences used were: GAPDH (forward: 5′-GGAGAAACCTGCCAAGTATGA-3′; reverse: 5′-GTGGGTGCAGCGAACTTTA-3′); IL-17 (forward: 5′-GCTGACCCCTAAGAAACCCC-3′; reverse: 5′-GTCCACAGAAAAACAAACACGA-3′); IFN-*γ* (forward: 5′-CGGCACAGTCATTGAAAGCCTA-3′; reverse: 5′-CTCTGCAGGATTTTCATGTCACC-3′); IL-4 (forward: 5′-TTCCAAGGTGCTTCGCATA-3′; reverse: 5′-TGCAGCTTATCGATGAATCCA-3′); IL-10 (forward: 5′-GCCTTATCGGAAATGATCCAGT-3′; reverse: 5′-GAAATCGATGACAGCGCCTC-3′); ROR*γ*t (forward: 5′-GGATGAGATTGCCCTCTACAC-3′; reverse: 5′-AGGAGGCCTTGTCGATGAG-3′); T-bet (forward: 5′-CCATTCCTGTCCTTCACCG-3′; reverse: 5′-CTGCCTTCTGCCTTTCCAC-3′); GATA-3 (forward: 5′-CTGGATGGCGGCAAAGC-3′; reverse: 5′-GTGGGCGGGAAGGTGAA-3′); Foxp3 (forward: 5′-AAGTACCACAATATGCGACCC-3′; reverse: 5′-GTAGGCGAACATGCGAGTAA-3′); MHCII (forward: 5′-TTACCAAGTACGGCAACATGACC-3′; reverse: 5′-AGATCTTCCAGTTCACGCCAT-3′); CD86 (forward: 5′-ACGCAAGCTTATTTCAATGGGA-3′; reverse: 5′-AAATAGTGCTCGTACAGAACCA-3′); CD80 (forward: 5′-TTGCCGTTACAACTCTCC-3′; reverse: 5′-GTTCTTATACTCGGGCCACA-3′); CD70 (forward: 5′-CGCCTGACATACCTGGTCCAC-3′; reverse: 5′-AGGGCATATCCACTGAACTCC-3′); ICOS-L (forward: 5′-ACACAACGGACAATAGCCTA-3′; reverse: 5′-GGAGAGCCACATTCTCTACGC-3′); PD-L1 (forward: 5′-GTCAATGCCCCATACCGCAAA-3′; reverse: 5′-TTCTCTTCCCACTCACGGGTT-3′); PD-L2 (forward: 5′-GCCTCTACCAGGTCACCAGT-3′; reverse: 5′-ACTTTGGGTTCCATCCGACT-3′); Ox40L (forward: 5′-ATTGACCTTCATTTCCGGGAG-3′; reverse: 5′-AGTATCAGGAGCATTTACAGT-3′); BTLA (forward: 5′-CCCCTTGAAGTTGGTCCTC-3′; reverse: 5′-TGTAGAACAGCTATACGACCC-3′); HVEM (forward: 5′-ATTCCTCATCTGCACGCGAAG-3′; reverse: 5′-CAGCAAACCCAACCTCGGTGA-3′); SLAM (forward: 5′-TCCCCTCCAGAGATTAAAGTGC-3′; reverse: 5′-TGTAAGTCACATGGTCCCCTT-3′); 4-1BBL (forward: 5′-AACAAGTTAGTGGACCGTTCCT-3′; reverse: 5′-GCTCCATGCAGATAAGCCCTCA-3′).

Cells were lysed in RIPA lysis buffer (10 mM Tris-HCL at pH 9.6, 1 mM EDTA, 150 mM NaCl, 1% NP-40, and 0.5 mM PMSF) supplemented with protease/phosphatase inhibitor. Proteins were denatured and electrophoresed in 8% Glycine-Tris/polyacrylamide gels and transferred to PVDF membranes. The membranes were blocked by TBST containing 5% BSA for 2 h and incubated in primary antibodies at 4°C overnight, labeled horseradish peroxidase conjugated antibodies and detected using a Fusion FX System. The blots were analyzed and quantified by Image J software.

### Histological Staining and Analyzing

After mice were sacrificed, the spinal cords were fixed with 10% neutral formalin, dehydrated and embedded in paraffin, and then cut into 5-mm sections. After drying at 42°C overnight, the spinal sections were dewaxed in xylol, rehydrated, stained with hematoxylin–eosin (H&E) and luxol fast blue (LFB), according to the manufacturers’ instructions. Tissue inflammation and demyelination were assessed by Image J pro software.

### Flow Cytometry

Spleen single cell suspension and cultured differentiated T cells were prepared after PBS washing; anti-mouse CD16/32 mAb (AF1460, Abcam, MA, USA) was used to block the non-specific binding. The combination of surface staining and intra-cellular cytokines staining was used in FACS. For surface makers’ staining, cells and fluorescent antibodies (for CD3, CD220, CD45, CD4, CD25) were incubated directly for 30 min at 4°C. Cells were fixed and permeabilized with a Fixation/Permeabilization Solution Kit (555028, BD Biosciences) for 20 min at room temperature before cytokine staining. For intracellular staining, specific fluorescent antibodies for cytokines (IFN-*γ*, IL-17, IL-4, IL-10) were incubated with cells for 30 min at 4°C. All FACS antibodies for flow cytometry were purchased from BD Biosciences and used according to the manufacturer’s instructions. Analysis was performed using a Beckman Coulter Cytoflex Flow Cytometer and FlowJo software.

### Statistical Analysis

Data are presented as the means ± SEM. Statistical analysis among groups was performed using one-way ANOVA with Tukey *post hoc* test. All statistical analyses were performed using Graph Pad Prism 7.0; p <0.05 was considered statistically significant.

## Results

### Th17 Responses Are Inhibited in Wild Type EAE Mice With One-Dose CRP Injection

**Figure 1:**
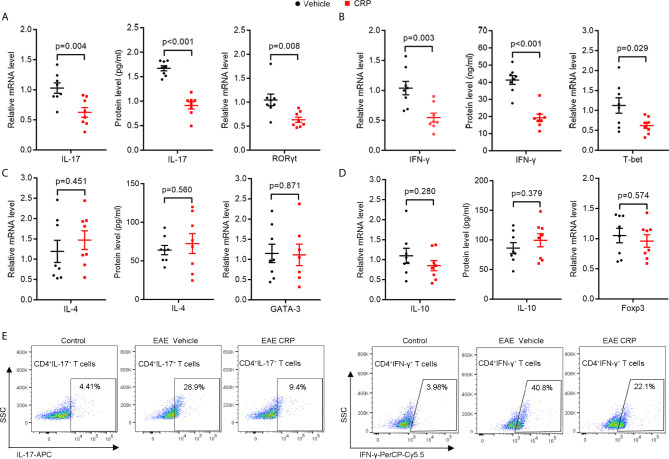
The Th17 responses are suppressed by one-dose CRP injection in WT EAE mice.Splenocytes were isolated form EAE and EAE CRP mice, then re-stimulated with 50 ug/ml MOG 24 h for qPCR, 72 h for ELISA and FACS. **(A)** Th17 relevant cytokine (IL-17) and transcription factor (ROR*γ*t) were examined by qPCR and ELISA (n = 8). **(B)** Th1 relevant cytokine (IFN-*γ*) and transcription factor (T-bet) were measured by qPCR and ELISA (n = 8). **(C)** Th2 relevant cytokine (IL-4) and transcription factor (GATA-3) were detected by qPCR and ELISA (n = 8). **(D)** Th2 relevant cytokine (IL-10) and transcription factor (Foxp3) were tested by qPCR and ELISA (n = 8). **(E)** Th17 (CD4^+^ IL-17^+^) and Th1 (CD4^+^ IFN-*γ* ^+^) cells were examined by FACS (n = 3). Data are presented as mean ± SEM; p < 0.05 was considered statistically significant.

To investigate whether CRP participates in Th17 response in EAE, WT C57BL/6 mice were immunized by myelin oligodendrocyte glycoprotein (MOG) in complete Freund’s adjuvantand pertussis toxin, with a single-dose injection of human CRP treatment. Unsurprisingly, the CRP-treated EAE mice showed a milder state of pathology than the vehicle-treated EAE mice (TBS-Ca2^+^), assessed by clinical score, body weight, HE, and LFB staining (**Supplementary Figure 1**), which is in agreement with published data (13, 16). We next collected the splenocytes to re-stimulate with MOG to evaluate the activation of CD4^+^ T cell subsets at the peak of EAE disease. The results showed that IL-17 and ROR*γ*t expression were high in EAE vehicle mice, while they were significantly decreased in CRP-treated EAE mice (**Figure 1A**). The IFN-*γ* and T-bet expression were also greatly reduced in CRP-treated EAE mice compared to their untreated littermates (**Figure 1B**), which is consistent with our previous findings (16). Meanwhile, there was no significant difference in the expression of IL-4 or GATA-3, genes relevant for Th2 function (**Figure 1C**); or in the expression of IL-10 or Foxp3, genes relevant for Treg function (**Figure 1D**). These data were further validated at a single cell level by flow cytometry analysis of Th17 and Th1 cells. As shown in **Figure1E**, CD4^+^ IL-17^+^ T cells and CD4^+^ IFN-*γ* ^+^ T cells were lower in CRP-treated EAE mice than in vehicle-treated EAE mice (CD4^+^ T cells were gated first), whereas Th2 and Treg subsets did not exhibit these changes and were always low in abundance (data not shown). These data indicated that Th17 responses were suppressed in MOG-induced EAE by CRP injection.

### CRP Suppresses the Th17 Responses Only in Splenocytes, But Not Isolated CD4^+^ T Cells

**Figure 2:**
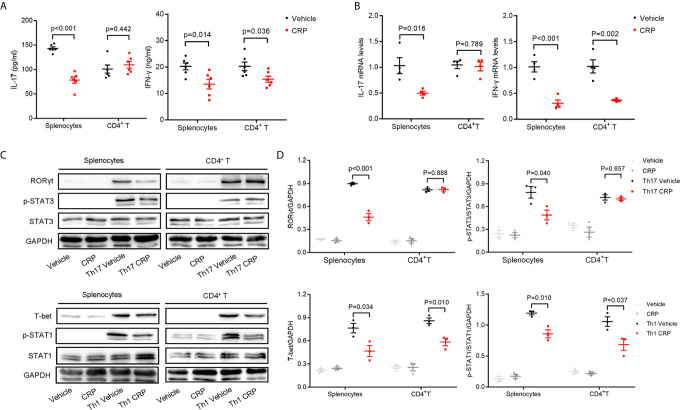
CRP reduces Th17 responses in splenocytes from WT mice.Splenocytes and CD4^+^ T cells were isolated from WT mice to distinguish the indirect and direct regulations of CRP on Th17. **(A)** The protein expression levels of IL-17 and IFN-*γ* were measured with CRP treatment in both splenocytes and CD4^+^ T cells by ELISA (n = 6). **(B)** The mRNA expression levels of IL-17 and IFN-*γ* were measured with CRP treatment in both splenocytes and CD4^+^ T cells by qPCR (n = 4). **(C)** Splenocytes and CD4^+^ T cells were cultured with CRP in Th17 and Th1 polarization conditions, then ROR*γ*t, T-bet, p-STAT-3, and p-STAT-1 were examined by WB. **(D)** The quantitative and statistical analysis of the WB results was presented (n = 3). Data are presented as mean ± SEM; p < 0.05 was considered statistically significant.

As we discussed in the *Introduction*, we hypothesized that CRP could participate in Th17 response indirectly in virtue of antigen presenting cells to shape T cell responses in EAE. However, it was too premature to say definitively which kind of APCs mediates this process. To verify our hypothesis and simulate the direct and indirect effects of CRP on Th17 response, we employed magnetic beads isolated CD4^+^ T cells and erythrocyte-lysed splenocytes, respectively, from WT mice to incubate with human CRP and vehicle. It should be emphasized that splenocytes contain not only all the kinds of APCs but also T cells; yet, since there was no direct effect of CRP on Th17 cells, the splenocytes served to model an indirect APC-mediated regulation of CRP on Th17. We first measured the expression of IL-17 after direct incubation of CRP with splenocytes and CD4^+^ T cells. These results showed that the level of IL-17 in the splenocytes group was obviously lower in CRP-treated samples than in vehicle samples, while in CD4^+^ T cell group, there was no change with CRP treatment. This finding was consistent in protein and gene levels (**Figures 2A, B** **)**. At the same time, the expression of IFN-*γ* was weakened with CRP treatment in both splenocytes and CD4^+^ T cells (**Figures 2A, B** **)**. To determine whether CRP affects the Th17/Th1 differentiation, we cultured the splenocytes and CD4^+^ T cells in Th17 and Th1 differentiation conditions to examine the expression of transcription factors and signal transducer and activator of transcription (STATs) by western blotting. Here, TGF-*β*, IL-1*β* and IL-6 were used to induce Th17 differentiation, while IL-12 was used to induce Th1 differentiation, then ROR*γ*t and p-STAT-3 were examined in Th17 differentiation, while T-bet and p-STAT-1 were examined in Th1 differentiation. ROR*γ*t and p-STAT-3 were down-regulated with CRP stimulation in the splenocytes, but not in CD4^+^ T cells (**Figures 2C, D** **)**. On the contrary, T-bet and p-STAT-1 expressions were substantially diminished in CRP-treated splenocytes and CD4^+^ T cells compared to vehicle (**Figures 2C, D** **)**. Collectively, our data clearly demonstrated that the Th17 response was attenuated indirectly by CRP treatment *in vitro* experiments, that is, with the help of APCs, but Th1 response could be regulated both directly and indirectly by CRP.

### Monocyte Derived CD11b^+^ CD11c^+^ DC Cells Mediate the Interaction of CRP on Th17 Response

**Figure 3:**
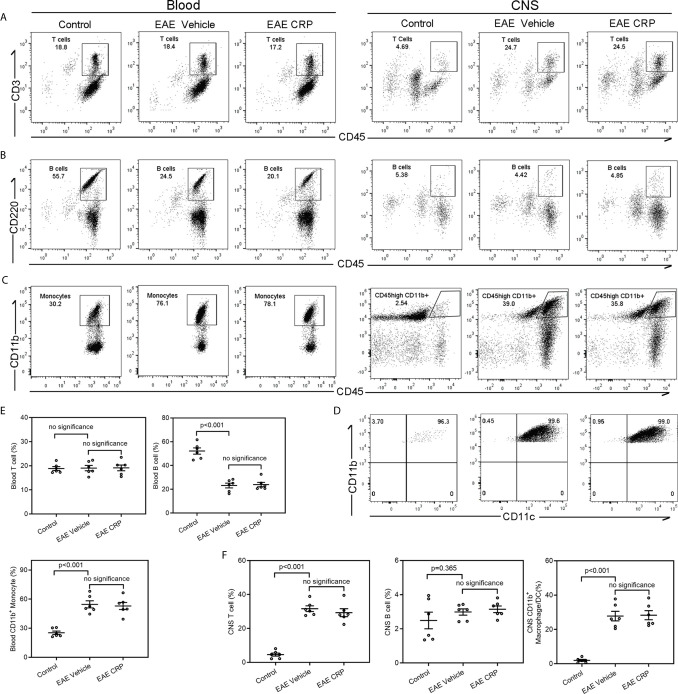
Analysis of immune cell composition of blood and CNS during the effector phase of EAE in WT mice.Flow cytometry of T cells **(A)**, B cells **(B)** and monocytes **(C)** in the blood and CNS from WT control mice, WT EAE vehicle mice and WT EAE CRP mice (n = 6). **(D)** The CNS-infiltrating CD45^high^ CD11b^+^ myeloid cells were proved to be CD11b^+^ CD11c^+^ DCs. **(E)** The quantitative and statistical analyses of the immune cells in blood were presented (n = 6). **(F)** The quantitative and statistical analyses of the immune cells in CNS were presented (n = 6). Data are presented as mean ± SEM; p < 0.05 was considered statistically significant. ns, no significance.

Immune cell infiltration is one of the core events in EAE development (35, 36). In addition to the encephalitogenic T cells, antigen presenting cells including B cells, macrophages, and DCs can infiltrate into the CNS and theoretically participate in T cell responses. Moreover, published reports have proposed that CRP influences the expression of chemokines and chemokine receptors, and thus participates in the migration and movement of immune cells into the CNS to modulate neuroinflammation (37, 38). To identify the candidate APCs that mediate the Th17 response by CRP and to see whether CRP affects the degree of immune cell infiltration to trigger Th17 response, the immune cell composition of blood and CNS from healthy mice (control), EAE mice and CRP-treated EAE mice were analyzed by flow cytometry. Our data revealed that compared to control mice, EAE mice maintained a similar percentage of T cells (CD45^+^CD3^+^ T cells) in the blood, whereas in CNS, T cells were increased from 5 to 25% of total immune cells in EAE mice (**Figures 3A, E, F** **)**. The percentage of B cells (CD45^+^B220^+^ B cells) was decreased from 55 to 25% in the blood, but they had no obvious difference in the CNS between control and EAE mice (**Figures 3B, E, F** **)**. Unexpectedly, the percentage of blood monocytes was raised from 30 to 80% in EAE mice compared to control mice, and in CD45^+^CD11b^+^ cells its level was elevated from 3 to 40% in the CNS during EAE (**Figures 3C, E, F** **)**. Further analysis showed that the population of CD45high CD11b^+^ cells were CD11b^+^CD11c^+^ DCs (**Figure 3D**). In addition to T cells, the majority of immune cells that infiltrated into the CNS were CD11b^+^ CD11c^+^ DCs in EAE mice. Notably, CRP treatment did not affect the ratio of T cells, B cells, and monocytes/macrophages/DCs in EAE mice. Therefore, it was plausible that moDCs may be the mediators of CRP-triggered Th17 response and that CRP did not alter the percentages of these immune cells in the blood and CNS.

### Fc*γ*R2B on moDCs Mediates the Regulation of CRP on Th17 Response

**Figure 4:**
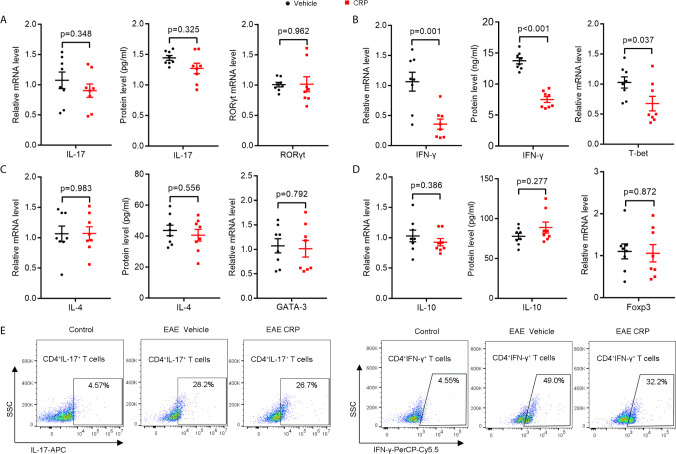
The decrease in Th17 response disappeared in Fc*γ*R2B^−/−^ EAE mice with CRP injection.**(A)** qPCR and ELISA analysis of IL-17 and ROR*γ*t expression in splenocytes and CD4^+^ T cells from Fc*γ*R2B^−/−^ EAE mice and Fc*γ*R2B^−/−^ EAE CRP (n = 8). **(B)** qPCR and ELISA analysis of IFN-*γ* and T-bet expression in splenocytes and CD4^+^ T cells from Fc*γ*R2B^−/−^ EAE mice and Fc*γ*R2B^−/−^ EAE CRP (n = 8). **(C)** qPCR and ELISA analysis of IL-4 and GATA-3 expression in splenocytes and CD4^+^ T cells from Fc*γ*R2B^−/−^ EAE mice and Fc*γ*R2B^−/−^ EAE CRP (n = 8). **(D)** qPCR and ELISA analysis of IL-10 and Foxp3 expression in splenocytes and CD4^+^ T cells from Fc*γ*R2B^−/−^ EAE mice and Fc*γ*R2B^−/−^ EAE CRP (n = 8). **(E)** Flow cytometry analysis of Th17 (CD4^+^ IL-17^+^) and Th1 (CD4^+^ IFN-*γ* ^+^) cells by cell surface and intercellular staining from Fc*γ*R2B^−/−^ EAE mice and Fc*γ*R2B^−/−^ EAE CRP (n = 3). Data are presented as mean ± SEM; p < 0.05 was considered statistically significant.

There are three major CRP receptors expressed on DCs, including Fc*γ*RI, Fc*γ*RII, and Fc*γ*RIII (39–41), but the Fc*γ*R2B is the only one that has been reported to mediate CRP function in EAE (27, 42). We were therefore interested in whether Fc*γ*R2B on moDCs could mediate the CRP function on Th17. We constructed the same EAE model using Fc*γ*R2B^−/−^ mice as we performed in WT mice and found that the pathology of Fc*γ*R2B^−/−^ EAE mice was slightly ameliorated with CRP injection as evidenced by clinical score, weight, and histochemistry analysis (**Supplementary Figure 1**), suggesting that CRP can regulate EAE not only by suppressing the response of Th1 cells, but also by an APCs independent mechanism. We next isolated the splenocytes for MOG re-stimulation from Fc*γ*R2B^−/−^ EAE mice with and without CRP treatment and found that the levels of IL-17 and ROR*γ*t expression were unaltered between these two groups (**Figure 4A**), which was opposed to the results obtained from WT mice (**Figure 1A**). However, IFN-*γ* and T-bet expression remained decreased in these two groups, which were consistent with WT mice (**Figures 4B** and **2B**). Also, there were no expression difference of IL-4, GATA-3, IL-10, and Foxp3 by MOG re-stimulation in the above two groups (**Figures 4C, D** **)**. In addition, the decrease of CD4^+^ IL-17^+^ T cells in WT mice disappeared in Fc*γ*R2B^−/−^ mice, but the CD4^+^ IFN-*γ* ^+^ T cells’ percentage was still reduced in Fc*γ*R2B^−/−^ mice as in WT mice (**Figure 4E**), CD4^+^ IL-4^+^ T cells, and CD4^+^ CD25^+^ Foxp3^+^ T cells had not detected the signals (data not shown).

**Figure 5:**
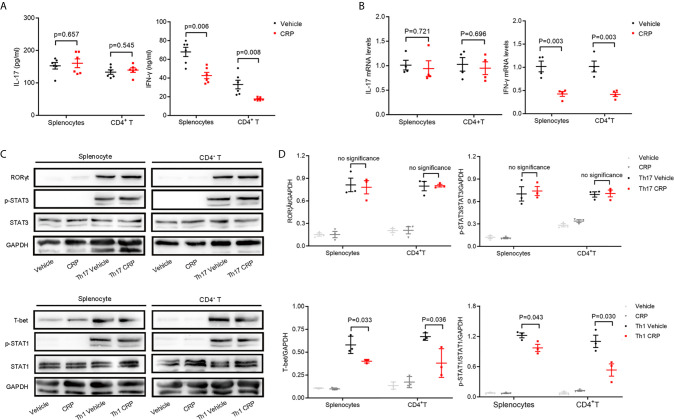
The decrease in Th17 response disappeared in Fc*γ*R2B^−/−^ splenocytes.Splenocytes and CD4^+^ T cells were isolated from Fc*γ*R2B^−/−^ mice to distinguish the indirect and direct regulations of CRP on Th17. **(A)** The protein expression levels of IL-17 and IFN-*γ* were measured in Fc*γ*R2B^−/−^ splenocytes and Fc*γ*R2B^−/−^ CD4^+^ T cells with or without CRP treatment (n = 6). **(B)** The mRNA expression levels of IL-17 and IFN-*γ* in Fc*γ*R2B^−/−^ splenocytes and Fc*γ*R2B^−/−^ CD4^+^ T cells with or without CRP treatment (n =4). **(C)** Fc*γ*R2B^−/−^ splenocytes and Fc*γ*R2B^−/−^ CD4^+^ T cells were isolated and incubated with or without CRP in Th17 and Th1 polarization condition, then ROR*γ*t, T-bet, p-STAT-3, and p-STAT-1 were detected by WB (n = 3). **(D)** The quantitative and statistical analysis of the WB results was presented (n = 3). Data are presented as mean ± SEM; p < 0.05 was considered statistically significant. ns, no significance.

Furthermore, splenocytes and CD4^+^ T cells were isolated from Fc*γ*R2B^−/−^ mice and incubated with or without CRP to look at whether Fc*γ*R2B mediates the CRP function on Th17 *in vitro* experiments. The ELISA analysis revealed that the IL-17 expression had no significant changes in splenocytes or CD4^+^ T cells with CRP stimulation from Fc*γ*R2B^−/−^ mice, while the IFN-*γ* expression was diminished in both splenocytes and CD4^+^ T cells in Fc*γ*R2B^−/−^ mice (**Figure 5A**). Further, qPCR analysis yielded similar results (**Figure 5B**). Moreover, we assessed the ROR*γ*t/T-bet and p-STAT-3/p-STAT-1 expressions under Th1 and Th17 polarization conditions in Fc*γ*R2B^−/−^ mice by WB. These data showed that ROR*γ*t and p-STAT-3 expression were unaltered with CRP treatment both in splenocytes and CD4+ T cells under Th17 polarization, whereas under Th1 polarization, the expressions of T-bet and p-STAT-1 remained down-regulated by CRP treatment both in splenocytes and CD4^+^ T cells (**Figure 5C**). Statistical and quantitative analysis of WB were shown in **Figure 5D**. Collectively, our data clearly demonstrated that Fc*γ*R2B mediated the function of CRP on Th17 response *in vivo* and *in vitro*.

### CRP Attenuates the Capability of Antigen Presentation of CNS Infiltrated moDCs Through Fc*γ*R2B

**Figure 6:**
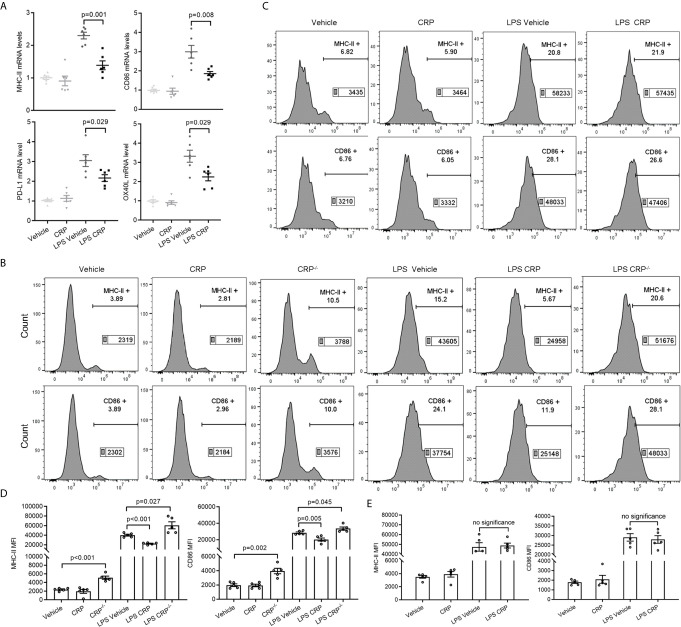
CRP diminishes the antigen presenting ability of moDCs though Fc*γ*R2B. **(A)** The mRNA expression levels of MHC-II, CD86, PDL-1, and OX40L were measured in LPS and LPS CRP samples from WT moDCs by qPCR (n = 6). **(B, D)** moDCs were established from WT mice and CRP^−/−^ mice, then incubated with or without CRP and LPS. The expression of MHC-II and CD86 was analyzed by flow cytometry and the MFI was recorded (n = 4). **(C, E)** moDCs were established from Fc*γ*R2B^−/−^ mice and cultured with or without CRP and LPS, then the expression of MHC-II and CD86 was analyzed by flow cytometry and the MFI was recorded (n = 4). Data are presented as mean ± SEM; p < 0.05 was considered statistically significant. ns, no significance.

We have proven that the increased monocytes in the blood infiltrate the CNS and differentiate into DCs during EAE development. Meanwhile, because we did not detect any percentage changes of these infiltrated immune cells caused by CRP treatment, so we speculated that CRP may reduce the antigen presentation ability of these monocyte derived DCs (moDCs) to participate in Th17 response and which could be mediated by Fc*γ*R2B expressed on moDCs. To test our speculation, moDCs were successfully isolated from PBMCs and established by GM-CSF and IL-4 as reported (43, 44), and LPS was added as a positive activator for antigen presentation molecules. Some crucial antigen presentation molecules were screened and verified in moDCs from WT mice by qPCR, including *MHC-II, CD86, CD80, CD70, COSL-1, PD-L1, PD-L2, OX40L, BTLA, HEVM, SLAM*, and *4-1BBL* (**Supplementary Figure 2A**), which were reported to be involved in modulating antigen presentation ability (45–47). The qPCR analysis showed that *MHC-II, CD86, PD-L1*, and *OX40L* had a lower expression in LPS CRP stimulation than LPS stimulation alone (**Figure 6A**). However, the difference in PD-L1 and OX40L expression was lost when we reexamined them by FACS (**Supplementary Figures 2B, C**). moDCs were next incubated with LPS from CRP^−/−^ mice and WT mice, and we found that the expression of MHC-II and CD86 was increased in CRP^−/−^ mice compared to moDCs with LPS from WT mice. Even with no LPS, moDCs from CRP^−/−^ mice had a higher expression of MHC-II and CD86 than from WT mice (**Figures 6B, D** **)**. Next, we used Fc*γ*R2B^−/−^ mice to further assess whether Fc*γ*R2B mediates the attenuation of antigen presentation ability of moDCs by CRP. The expression of MHC-II and CD86 had no apparent difference in LPS and CRP-treated samples compared to LPS alone (**Figures 6C, E** **)**. Taken together, these results indicated that CRP attenuated the antigen presentation ability of CNS infiltratory moDCs by inhibiting the expression of MHC-II and CD86, and this process was mediated by Fc*γ*R2B expressed on moDCs.

### NF-*κ*B and ERK Signaling Pathways Involve in Suppressing the Expression of MHC-II and CD86 in moDCs by CRP

**Figure 7:**
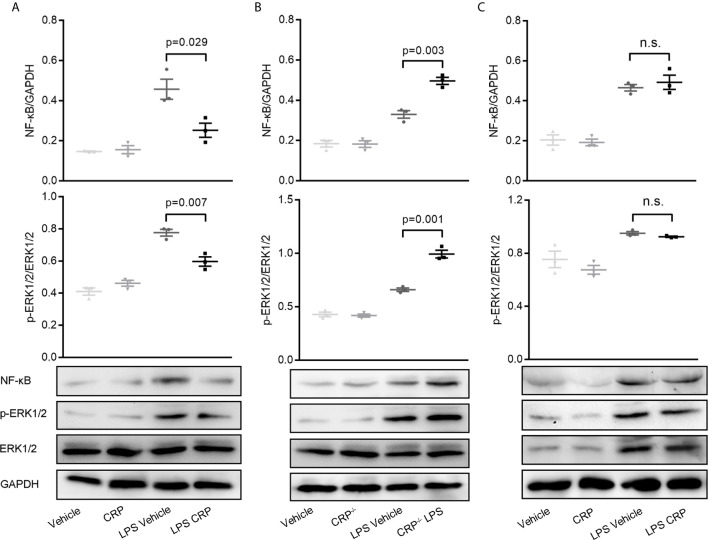
NF-*κ*B and ERK signaling is involved in decreasing the antigen presenting ability of moDCs by CRP.**(A)** Western Blot and quantitative analyses of NF-*κ*B and p-ERK in moDCs from WT mice with or without LPS and CRP (n = 3). **(B)** Western Blot and quantitative analyses of NF-*κ*B and p-ERK in moDCs from WT mice and CRP^−/−^ mice with or without LPS and CRP (n = 3). **(C)** Western Blot and quantitative analyses of NF-*κ*B and p-ERK in moDCs from Fc*γ*R2B^−/−^ mice with or without LPS and CRP (n = 3). Data are presented as mean ± SEM; p < 0.05 was considered statistically significant.

Many previous studies have substantiated unequivocally that NF-*κ*B is a crucial regulator of DCs for antigen presentation and therefore is involved in DC maturation (23, 48, 49). ERK is a another signaling molecule that contributes to DCs’ survival and maturation through increasing the TNF-α production (50). To delineate the signaling pathways underlying the observed effects of CRP on antigen presentation ability of DCs *via* Fc*γ*R2B, moDCs were established from WT mice, CRP^−/−^ mice, and Fc*γ*R2B^−/−^ mice, then the expressions of NF-*κ*B (p65) and ERK were evaluated by WB. In WT mice, WB and quantitative analysis showed a significant decrease of NF-*κ*B and phosphorylated ERK in LPS and CRP stimulated samples when compared to LPS stimulation alone, but there was no difference in vehicle and CRP samples (**Figure 7A**). Opposite results were obtained by using moDCs from WT mice and CRP^−/−^ mice; a higher NF-*κ*B and phosphorylated ERK expression in CRP^−/−^ mice than in WT mice (**Figure 7B**) indicated that CRP played an important role in maintaining the moderate activation of antigen presenting cells. In Fc*γ*R2B^−/−^ mice, the NF-*κ*B and phosphorylated ERK did not differ significantly between LPS and LPS CRP samples (**Figure 7C**), presumably because the absence of Fc*γ*R2B prevented CRP from interfering with DCs’ antigen presentation. Overall, our results indicated that NF-*κ*B and ERK signaling were involved in Fc*γ*R2B-mediated effects of CRP on antigen presentation and Th17 response.

## Discussion

In a previous study, we reported that CRP modulates Th1 and Th2 responses directly by binding to naïve T cells, but there was no effect observed on Th17 response at that time. In this paper, we focused on whether CRP participates in the Th17 response and explored the indirect regulation of CRP on Th17 *via* APCs. Initially, the MOG-induced EAE model was utilized to prove the Th17 response was suppressed *in vivo* with CRP injection, then we used splenocytes and CD4^+^ T cells to distinguish the difference in Th17 and Th1 response *in vitro*, further speculating that a potential possible of CRP is to act on Th17 response indirectly through APCs. Secondly, we analyzed the immune cell composition in the blood and CNS during the peak of EAE and found that apart from T cells, the moDCs were the main immune cells infiltrating into the CNS, which provides the possibility of CRP to participate in Th17 regulation. Thirdly, Fc*γ*R2B had been reported to have a crucial role in EAE regulation of CRP, so Fc*γ*R2B^−/−^ mice were used to verify that CRP participates in Th17 response regulation by Fc*γ*R2B *in vivo* and *in vitro*. Finally, the antigen presenting molecules were screened in moDCs from WT mice, which could be influenced by CRP treatment, and NF-*κ*B and ERK signaling was proved to be involved in this process.

Although many previous studies have reported the regulatory role of CRP on normal DCs (27, 42, 51, 52), the studies of CRP on monocyte derived DCs were limited and controversial (24, 28), and their concern with DCs was only because the DCs could express the CRP receptors, but in our study, we focused on DCs because we had solid data to demonstrate that moDCs are the main immune cells infiltrating the CNS to elicit an indirect T cell response in EAE. Moreover, we concentrated on the regulation of CRP on Th17 in EAE; this is very different from previous studies. Our data showed clearly that Th17 and Th1 responses were impaired when the antigen presenting molecule expressions were inhibited by CRP; previous studies did not investigate CD4^+^ T cell subtypes, but instead only evaluated T cell proliferation. Furthermore, we have more comprehensively explored the mechanism. WT mice, CRP^−/−^ mice, and Fc*γ*R2B^−/−^ mice were used to demonstrate the importance of CRP on the antigen presenting ability of moDCs, and finally NF-*κ*B and ERK signaling and the Fc*γ*R2B receptor were confirmed to be involved in this process.

**Figure 8:**
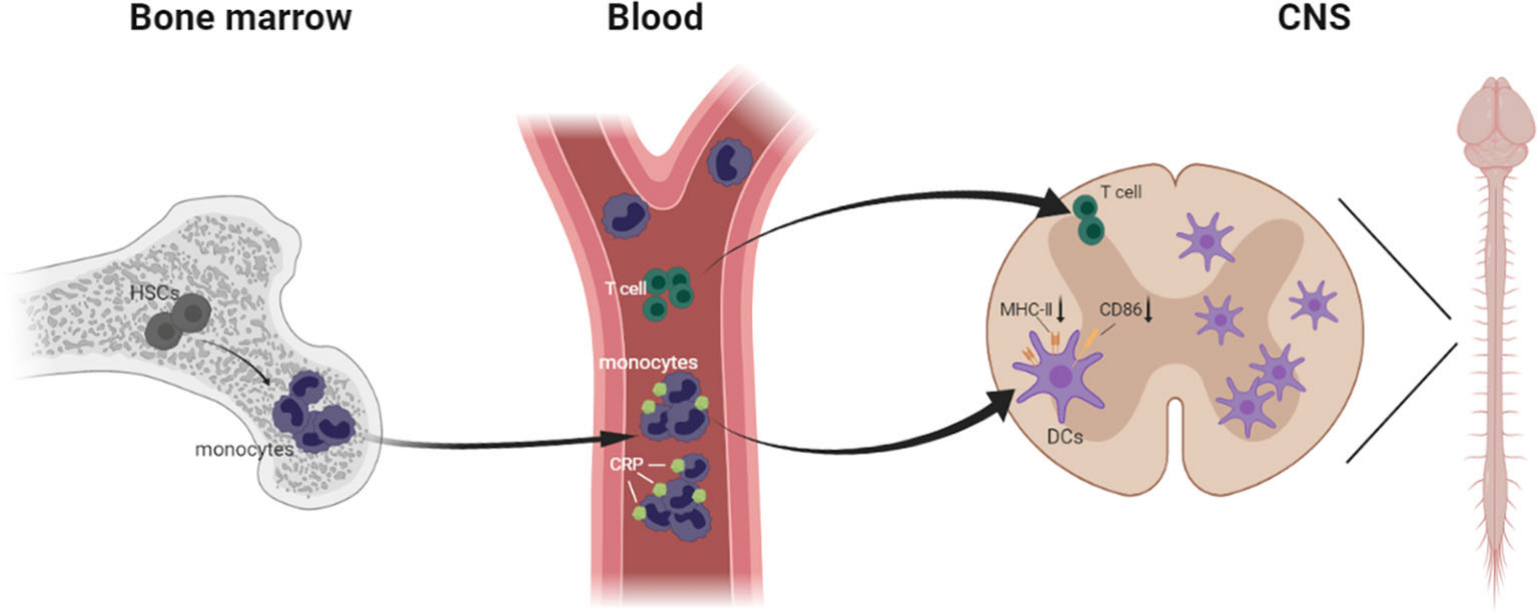
Schematic model demonstrating immune cells infiltrating from the bone marrow to the CNS in EAE.

Most important, we verified that moDCs are the main antigen presenting cells in EAE, and we described a precise pathway for inflammatory immune cell infiltration during EAE development, from bone marrow hematopoietic stem cells to blood monocytes to CNS DCs. More concretely, when the mice were immunized with MOG in complete Freund’s adjuvant, the blood monocytes were increased sharply and implicated as essential players in defense against microbial pathogens; then they were activated and differentiated into macrophages and DCs, infiltrating the CNS. Meanwhile, in CRP-treated mice, CRP was injected intraperitoneally into mice and absorbed into the blood through the capillaries, where these immune cells could be primed by CRP, thereby influencing monocyte differentiation and maturation. Eventually these immune cells infiltrate the CNS and trigger specific CD4^+^ T cell responses. Nevertheless, these circulating blood monocytes usually descend from self-renewing hematopoietic stem cells that initiate myeloid differentiation (53, 54) (**Figure 8**).

During all the data collection, Th17 and Th1 relevant data were always harvested together, because both Th1 and Th17 subsets are the main mediators of EAE (55), and we wanted to know all the contributions of CRP on EAE remission. Moreover, we proposed that the regulation of CRP on Th17 requires the aid of APCs, while for Th1 modulation, direct and indirect regulations work together, so there is a difference of CRP on Th17 and Th1 responses. Furthermore, we should note that the indirect regulation is not specific to Th17 response, but also affects Th1 and Th2 responses, depending on the disease state and the immune microenvironment at that time. In this regard, all T cell mediated autoimmune diseases like SLE and RA could theoretically be improved by CRP with the help of APCs. Overall, the indirect APC-mediated pathway presented in this paper enriches our understanding of how CRP regulates T cells and leads to EAE remission.

In conclusion, although CRP is an innate molecule, more and more studies focus on its acquired immune function in recent years. Our present work expands on the existing research, and we put forward a new way for CRP participating in the regulation of Th17 response in EAE, an effect which depends on the APCs and is mediated through Fc*γ*R2B, as well as NF-*κ*B and ERK signaling pathways. Our study adds a new dimension to understand the multi-faceted effects of CRP in EAE remission, which suggests that CRP may be a novel drug target for the fundamental prevention and treatment of MS and other T-cell mediated autoimmune diseases.

## Data Availability Statement

The raw data supporting the conclusions of this article will be made available by the authors, without undue reservation.

## Ethics Statement

The animal study was reviewed and approved by the Experimental Animal Center of Xi’an Jiaotong University.

## Author Contributions

LZ supervised the project. LZ and YZ designed the research. Z-YS and YZ performed most of the experiments. KW, WL, M-JG, FW, and YZ carried out part of the experiments. LZ, Z-YS, and MP analyzed the data and drafted the manuscript. All authors contributed to the article and approved the submitted version.

## Funding

This work was supported by the following grants: National Natural Science Foundation of China (Nos. 82070876, 31700685, 31800852), Scientific Research Fund of Xi’an Jiaotong University (No. XJJ2018126), China Postdoctoral Science Foundation (No. 2020M673421), and Project of Shaanxi Key Research and Development Plan (No. 2020SF-082).

## Conflict of Interest

The authors declare that the research was conducted in the absence of any commercial or financial relationships that could be construed as a potential conflict of interest.

## Supplementary Material

Supplementary Figure 1 CRP alleviates MOG-immunized EAE severity both in WT mice and FcγR2B-/- mice. **(A)** The clinical sores of EAE were recorded daily in WT mice and FcγR2B-/- mice respectively. **(B)** The body weight was recorded daily in WT mice and FcγR2B-/- mice, which is consistent with EAE severity. **(C)** The degree of demyelination of each group was shown in LFB staining. **(D)** HE staining displayed the infiltration of immune cells for each groups. Data are presented as mean ± SEM, p < 0.05 was considered statistically significant.

Supplementary Figure 2 CRP effects the antigen presenting ability of moDCs from WT mice. **(A)** Antigen presenting molecular MHC-II, CD86, CD80, CD70, COSL-1, PD-L1, PD-L2, OX40L, BTLA, HEVM, SLAM and 4-1BBL were screened by qPCR (n = 6). **(B)** Flow cytometry of PD-L1 was no apparent difference between LPS and LPS CRP treated samples (n = 4). **(C)** Flow cytometry of OX40l was unchanged between LPS and LPS CRP treated samples (n = 4). Data are presented as mean ± SEM, p < 0.05 was considered statistically significant.
