# Supplementary figures and images for: C-Reactive Protein Suppresses the Th17 Response Indirectly by Attenuating the Antigen Presentation Ability of Monocyte Derived Dendritic Cells in Experimental Autoimmune Encephalomyelitis
Source: Front Immunol. 2021 Mar 25;12:589200. doi: 10.3389/fimmu.2021.589200 (PMC8027258; doi:10.3389/fimmu.2021.589200)

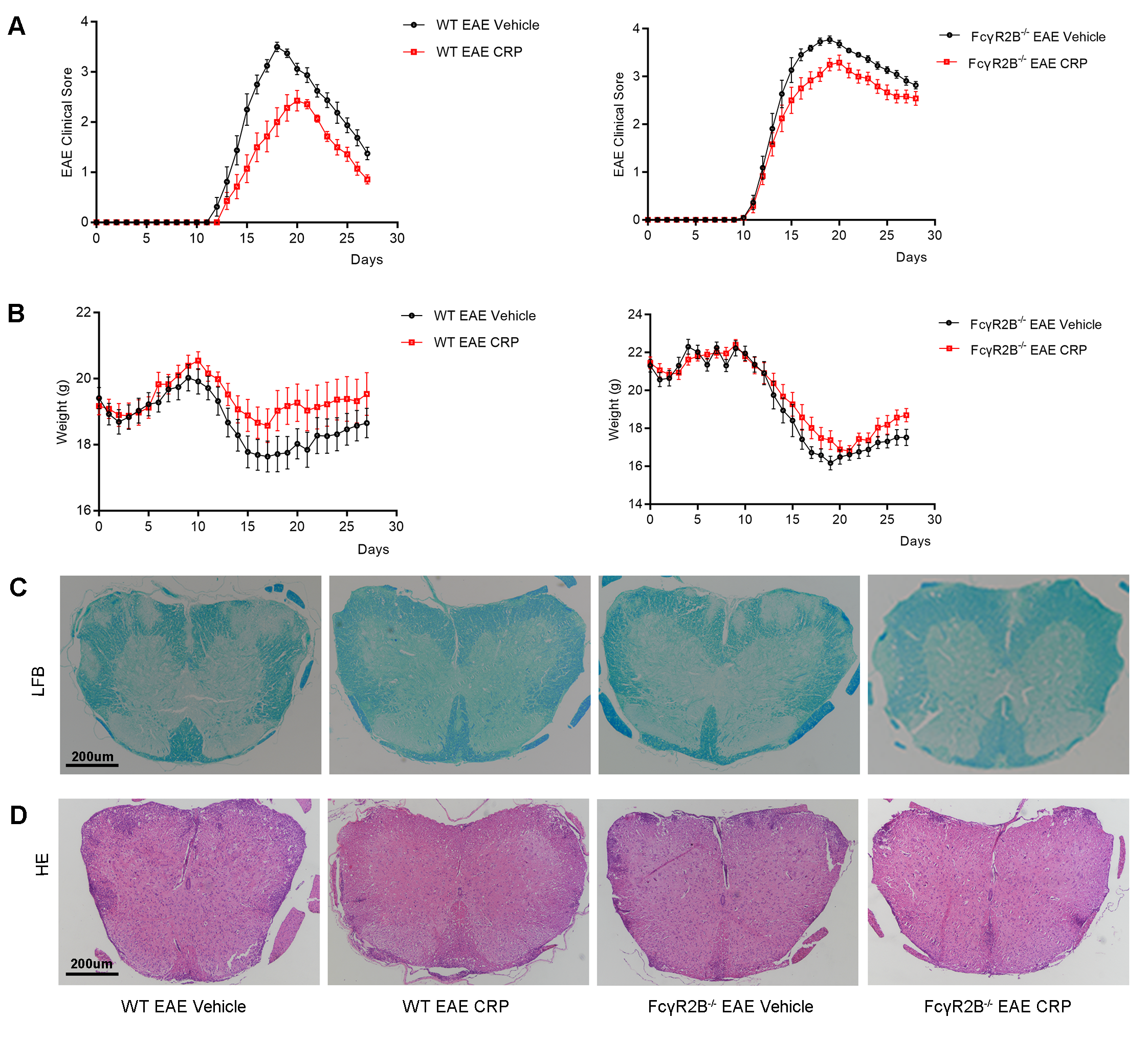

Supplement: Supplementary Figure 1 — CRP alleviates MOG-immunized EAE severity both in WT mice and FcγR2B-/- mice. (A) The clinical sores of EAE were recorded daily in WT mice and FcγR2B-/- mice respectively. (B) The body weight was recorded daily in WT mice and FcγR2B-/- mice, which is consistent with EAE severity. (C) The degree of demyelination of each group was shown in LFB staining. (D) HE staining displayed the infiltration of immune cells for each groups. Data are presented as mean ± SEM, p < 0.05 was considered statistically significant. [file Image_1.tif]

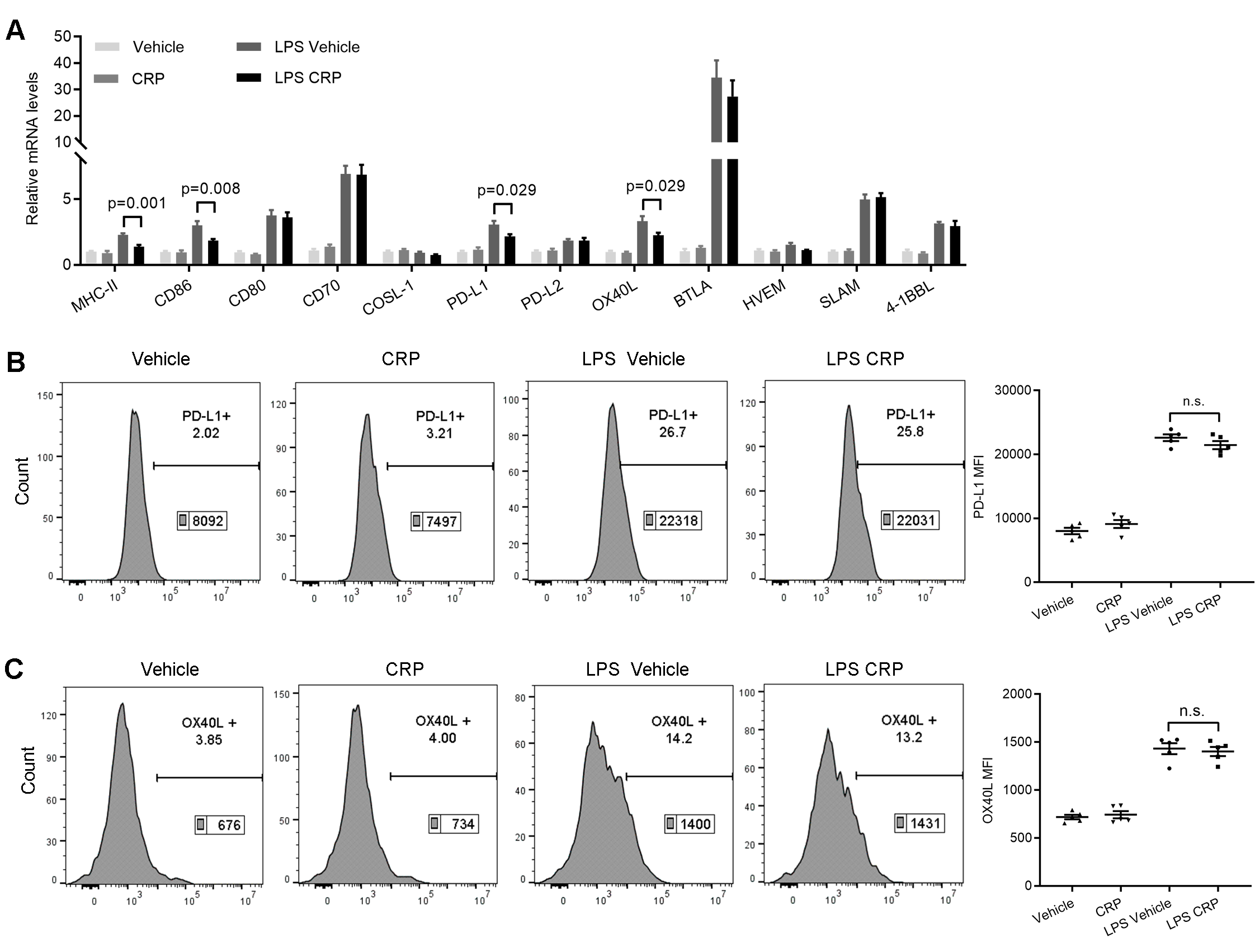

Supplement: Supplementary Figure 2 — CRP effects the antigen presenting ability of moDCs from WT mice. (A) Antigen presenting molecular MHC-II, CD86, CD80, CD70, COSL-1, PD-L1, PD-L2, OX40L, BTLA, HEVM, SLAM and 4-1BBL were screened by qPCR (n = 6). (B) Flow cytometry of PD-L1 was no apparent difference between LPS and LPS CRP treated samples (n = 4). (C) Flow cytometry of OX40l was unchanged between LPS and LPS CRP treated samples (n = 4). Data are presented as mean ± SEM, p < 0.05 was considered statistically significant. [file Image_2.tif]
